# Exposure to ambient air pollution and calcification of the mitral annulus and aortic valve: the multi-ethnic study of atherosclerosis (MESA)

**DOI:** 10.1186/s12940-017-0346-x

**Published:** 2017-12-21

**Authors:** Martin Tibuakuu, Miranda R. Jones, Ana Navas-Acien, Di Zhao, Eliseo Guallar, Amanda J. Gassett, Lianne Sheppard, Matthew J. Budoff, Joel D. Kaufman, Erin D. Michos

**Affiliations:** 1 Ciccarone Center for the Prevention of Heart Disease, Johns Hopkins School of Medicine, Baltimore, MD USA; 2 Department of Medicine, St. Luke’s Hospital, Chesterfield, MO USA; 3 Department of Epidemiology, Johns Hopkins Bloomberg School of Public Health, Baltimore, MD USA; 4 Department of Environmental Health Sciences, Columbia University School of Public Health, New York, NY USA; 5 Department of Environmental and Occupational Health Sciences, University of Washington, Seattle, WA USA; 6 Department of Biostatistics, University of Washington, Seattle, WA USA; 7 Division of Cardiology, Harbor-UCLA Medical Center, Los Angeles, CA USA; 8 Department of Epidemiology, University of Washington, Seattle, WA USA; 9 Division of Cardiology, Johns Hopkins School of Medicine, Blalock 524-B, 600 N. Wolfe Street, Baltimore, MD 21287 USA

**Keywords:** Air pollution, Valvular calcification, Aortic valve calcification, Mitral annulus calcification, Epidemiology, Prevention

## Abstract

**Background:**

Long-term exposure to high ambient air pollution has been associated with coronary artery calcium (CAC), a marker of cardiovascular disease (CVD). Calcifications of left-sided heart valves are also markers of CVD risk. We investigated whether air pollution was associated with valvular calcification and its progression.

**Methods:**

We studied 6253 MESA participants aged 45–84 years who underwent two cardiac CT scans 2.5 years apart to quantify aortic valve calcium (AVC) and mitral annular calcium (MAC). CAC was included for the same timeframe for comparison with AVC/MAC. Ambient particulate matter <2.5 μm (PM_2.5_) and oxides of nitrogen (NO_x_) concentrations were predicted from residence-specific spatio-temporal models.

**Results:**

The mean age (SD) of the study sample was 62 (10) years, 39% were white, 27% black, 22% Hispanic, and 12% Chinese. The prevalence of AVC and MAC at baseline were 13% and 9% respectively, compared to 50% prevalence of CAC. The adjusted prevalence ratios of AVC and MAC for each 5 μg/m^3^ higher PM_2.5_ was 1.19 (95% CI 0.87, 1.62) and 1.20 (0.81, 1.77) respectively, and for CAC was 1.14 (1.01, 1.27). Over 2.5 years, the mean change in Agatston units/year for each 5 μg/m^3^ higher PM_2.5_ concentration was 0.29 (−5.05, 5.63) for AVC and 4.38 (−9.13, 17.88) for MAC, compared to 8.66 (0.61, 16.71) for CAC. We found no significant associations of NOx with AVC and MAC.

**Conclusion:**

Our findings suggest a trend towards increased 2.5-year progression of MAC with exposure to outdoor PM_2.5_, although this association could not be confirmed. Additional well-powered studies with longer periods of follow-up are needed to further study associations of air pollution with valvular calcium.

**Trial registration:**

Although MESA is not a clinical trial, this cohort is registered at ClinicalTrials.gov Identifier: NCT00005487; Date of registration May 25, 2000.

## Background

Several epidemiologic studies have associated long-term exposure to air pollution with increased cardiovascular morbidity and mortality [1–3]. A possible underlying mechanism is through increased pulmonary and systemic inflammation [4, 5]. Additionally, exposure to ambient air pollution has been linked to increased risk for some traditional atherosclerotic cardiovascular disease (CVD) risk factors, including diabetes, hypertension and dyslipidemia [6–8]. Prior work from the Multi-Ethnic Study of Atherosclerosis and Air Pollution (MESA Air) reported an association of increased exposure to air pollution with progression of coronary artery calcification (CAC) [9], a powerful subclinical marker of absolute and relative CVD risk [10, 11]. Similar to CAC, calcification involving left-sided heart valves (mitral annular calcification [MAC] and aortic valve calcification [AVC]) are more prevalent among patients with coronary artery disease [12]. Although CAC and calcification involving left-sided heart valves are located at different territories of the cardiovascular system, they are strongly associated [13] and have been shown to share similar traditional CVD risk factors [12, 14, 15]. When severe, AVC and MAC can lead to valvular dysfunction, which may lead to heart failure. Also, asymptomatic cases of AVC and MAC have been linked to increased risk for myocardial infarction, stroke, atrial fibrillation and vascular death, independent of traditional CVD risk factors [16, 17]. Whether exposure to air pollution is also associated with AVC and MAC is unknown.

We sought to examine associations of household-level concentrations of particulate matter less than 2.5 μm in diameter (PM2.5) and oxides of nitrogen (NOx) with the prevalence and progression of AVC and MAC measured by cardiac computed tomography (CT), and compared to CAC, in a well-characterized cohort from six metropolitan areas in the United States (U.S.).

## Methods

### Study population

Detailed description of MESA has been published elsewhere [18]. In brief, 6814 White, Black, Hispanic and Chinese participants aged 45–84 years and free of clinical CVD at time of enrollment were recruited from six U.S. cities (Baltimore, Maryland; Chicago, Illinois; Forsyth County, North Carolina; Los Angeles, California; New York, New York and St. Paul, Minnesota). The first enrollment took place between 2000 and 2002. Follow-up visits took place between 2002 and 2004 for Exam 2 and 2004–2005 for Exam 3. MESA was designed to investigate the significance of subclinical CVD. MESA Air was an ancillary study of MESA designed to examine the impact of individual-level estimates of air pollution on CVD risk [19]. Institutional Review Boards of all participating sites approved the study, and all participants signed informed consent.

**Fig. 1:**
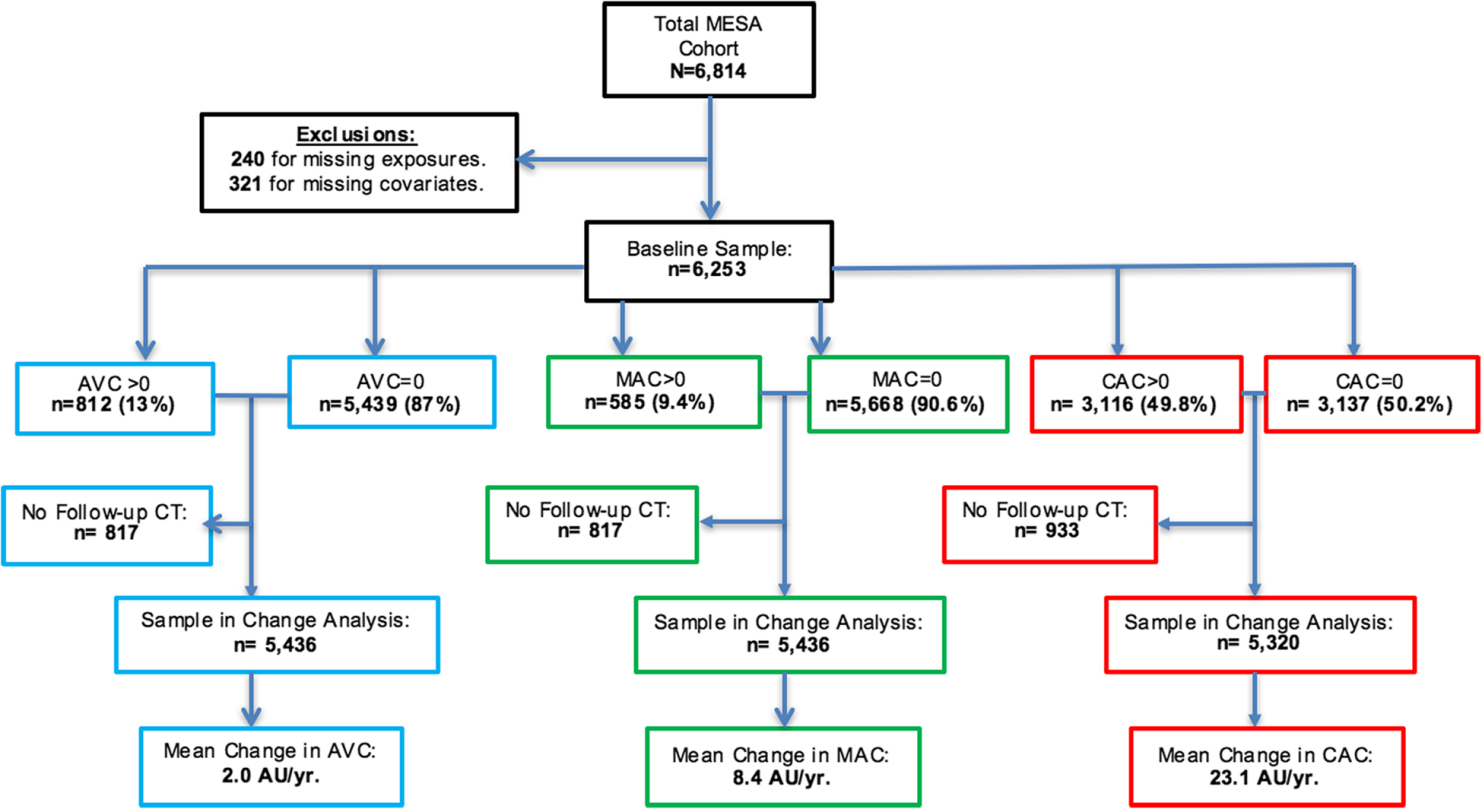
A flow diagram illustrating the number of participants included in prevalence analysis at baseline and progression analyses after a mean follow-up of 2.5 years

Of the 6814 participants enrolled in MESA, 240 participants were excluded for missing year 2000 air pollution concentrations and 321 for missing key covariates, leaving a total of 6253 participants in our baseline analytic sample (Fig. 1).

### Measurement of concentrations of air pollutants (PM_2.5_ and NO_x_)

MESA Air predicted outdoor (household-level) concentrations of particulate matter less than 2.5 μm in diameter (PM_2.5_) and oxides of nitrogen (NO_x_) for MESA participants using residence-specific hierarchical spatiotemporal models as previously described [19–21]. First, observed outdoor concentrations of pollutants were measured from nearly 100 consecutive fortnightly air samples collected from 27 fixed site monitors placed at MESA communities by MESA Air to supplement data from the US Environmental Protection Agency (EPA) Air Quality System. To create a spatially rich data for NO_x_, nearly 100 simultaneous fortnightly saturation samples were also collected from all MESA cities on three occasions (“snapshots”) to capture seasonal variations. Residence-specific hierarchical spatio-temporal models accounting for irregular monitoring and using all monitoring data (EPA’s monitoring data and data from MESA Air’s fixed monitors) were then developed to predict household-level PM_2.5_ and NO_x_ for each fortnight over the course of the study [21].

For progression analysis, averages of fortnightly residence-specific predictions of pollutants from baseline CT to follow-up CT scan dates, rounded to the nearest whole year were used to account for potential seasonal effects. Year 2000 concentrations were used in our baseline prevalence analysis. Additionally, in secondary analyses, individually-weighted exposure to PM_2.5_ (PM_2.5iwa_) for each fortnight over the course of the study was calculated by integrating outdoor PM_2.5_, indoor infiltration and time-location data [22]. Indoor infiltration was estimated using meteorology and responses from MESA Air participants regarding heating choices, air conditioner use, and window opening [23].

### Measurement of AVC and MAC

AVC and MAC were assessed by cardiac-gated electron-beam cardiac CT at three study centers and a four-slice multidetector row helical CT at the other 3 centers. The MESA scanning protocol has been previously published [24]. Briefly, participants underwent two consecutive scans at the same visit and results were averaged to enhance the accuracy of calcium assessments. AVC and MAC were quantified by the Agatston scoring method [25]. All studies were interpreted at one central reading center (Harbor-UCLA Research and Education Institute, Los Angeles, CA). Any detectable calcium was defined as a score > 0 Agatston units (AU). A minimum focus of calcification was based on at least 4 contiguous voxels, resulting in identification of calcium of 1.15 mm^3^ with the multi-detector row helical CT scanners (0.68 Å~ 0.68 Å~ 2.50 mm) and 1.38 mm^3^ with the electron beam CT scanners (0.68 Å~ 0.68 Å~ 3.00 mm). Details of the image acquisition and interpretation protocols, quality control measures and inter-observer reliability characteristics have been reported [24, 26]. Per study protocol, all participants underwent a cardiac CT scan at baseline, a random half at Exam 2 and the other half at Exam 3. Although some MESA Air participants additionally underwent a third cardiac CT at Exam 5 (2010–2012) for CAC as previously described [9], these latter scans have not yet been interpreted for MAC and AVC. Thus, cardiac CT data from Exam 1 and Exams 2/3 were used for this analysis.

### Covariates

Demographics, behavioral risk factors, medical history and medication history were obtained using standardized questionnaires at Exam 1. The following variables were adjusted for in our analyses: age, sex, race/ethnicity (White; Chinese; Black; or Hispanic), education (less than high school; high school or vocational school; college, graduate or professional school), study site, scanner type, annual family income (categorized as $24,999 or less; $25,000 to $49,999; $50,000 to $74,999; and $75,000 or greater), cigarette smoking status (categorized as current; former; never), second-hand smoke exposure (in hours per week) and physical activity level (METS*minutes/week of moderate or vigorous activity). We also included baseline CVD risk factors: family history of heart attack, systolic blood pressure, high-sensitivity C-reactive protein (CRP), diabetes (defined as fasting blood glucose ≥126 mg/dl, or nonfasting glucose ≥200 mg/dl or medication use), body mass index (BMI, as a continuous variable), estimated glomerular filtration rate (eGFR) derived from the CKD-EPI eq. [27], total cholesterol, HDL-cholesterol, and medication usage (lipid-lowering therapy or antihypertensive).

### Statistical analysis

Since recent epidemiologic studies have suggested that separate biological mechanisms may explain AVC and MAC [28, 29], we opted to model these as separate outcomes in this study. Since Air Quality Standards are set based on outdoor concentrations of pollutants, associations of outdoor-levels of PM_2.5_ and NO_x_ with AVC and MAC were the primary focus of this study. PM_2.5iwa_ was included as a secondary analysis. Prevalent AVC and MAC were defined as Agatston score > 0 at baseline. Modified Poisson regression model with robust variance estimation was then used to estimate the adjusted prevalence risk ratio (adjPRR) with 95% confidence intervals (CI) for each 5 μg/m^3^ higher PM_2.5_ concentration (outdoor and PM_2.5iwa_) and 40 ppb higher in outdoor NO_x_ [30]. Each pollutant (PM_2.5_ or NO_x_) was included in separate models for each primary outcome (AVC or MAC), and parameters were estimated as 5 μg/m^3^ and 40 ppb higher because they are close to the interquartile ranges of PM_2.5_ and NO_x_ respectively [9].

For progression of AVC and MAC, we subtracted baseline Agatston scores from follow-up scores for all participants with baseline and follow-up CT data (Fig. 1), and divided the difference by time between CT scans to obtain an annual change in AVC and MAC from baseline. Staged linear regression models were used to estimate associations of PM_2.5_ and NO_x_ (in separate models) with annual change in AVC or MAC from baseline.

Models were staged to be similar to the prior published analyses about air pollution and CAC [9] as follows: Model 1 adjusted for age, sex, race/ethnicity, study site and CT scanner type; Model 2 additionally adjusted for smoking status, second-hand smoke exposure, physical activity, BMI, total and HDL cholesterol and statin use; Model 3 (our main Model) additionally adjusted for income and education; and Model 4 additionally adjusted for the CVD risk factors of family history of heart attack, diabetes, systolic blood pressure, use of antihypertensive medication, eGFR and CRP.

Although the association of air pollution with 10-year change in CAC from the MESA Air Study has previously been published [9], in secondary analyses, we replicated the above analyses using CAC data up to MESA Exam 3 (an average follow-up of 2.5 years and the same time frame for the available data for AVC and MAC) to enable comparison with our AVC and MAC findings. Also, multiplicative interaction terms were created to evaluate for effect modification by race/ethnicity, age, sex and by the other pollutant (PM_2.5_ or NO_x_).

All statistical analyses were performed using STATA 13 (StataCorp LP, College Station, TX) and significance was considered at *P* value of 0.05 or less.

## Results

Table 1 summarizes the demographic, general health characteristics and air pollution exposures of the analytical sample (*n* = 6253) at baseline, by prevalent AVC and MAC. The mean (standard deviation) age of the cohort was 62 (10) years and included 39.4% Whites, 11.9% Chinese, 26.5% Blacks, and 22.2% Hispanics. Compared to those without AVC, participants with AVC at baseline were older [70 (8) vs. 61 (10) years], more likely to be men [59.8% vs. 45.4%], have higher systolic blood pressures [135 (22) vs. 125 (21) mmHg] and less likely to be physically active [3480 (4890) vs. 4151(5689) MET-minutes/week]. Similarly, when compared to those without MAC, participants with prevalent MAC at baseline were older, more likely to have higher systolic blood pressures and less likely to be physically active. Of note, participants with prevalent AVC and MAC at baseline were less likely to be current smokers, but on the other hand, these participants were older, more likely to be on cardiac medications and thus more likely to have quit smoking.

**Table 1:** Baseline (2000–2002) characteristics of study participants by prevalent AVC and MAC (*N* = 6253)

	All	AVC at Baseline		MAC at Baseline	
	(*N* = 6253)	Yes (*n* = 812, 13%)	No (*n* = 5441, 87%)	Yes (*n* = 585, 9%)	No (*n* = 5668, 91%)
Characteristic					
Demographics					
Age (years)	62 ± 10	70 ± 8	61 ± 10†	72 ± 8	61 ± 10†
Men (%)	47.2	59.8	45.4†	40.6	47.9†
Ethnicity (%)					
White	39.4	45.6	38.1†	49.1	38.4†
Chinese	11.9	7.9	12.9	5.8	12.5
Black	26.5	23.1	26.8	20.4	27.1
Hispanic	22.2	23.4	22.3	24.7	22
Education level (%)					
Less than high school	17.6	23.2	17.0†	22.8	17.0†
High school or vocational school	41.4	41.5	41.2	43.2	41.2
College, graduate or professional school	41.1	35.3	41.8	34.1	41.8
Annual family income (in $, %)					
≤24,999	30.9	38.1	29.9	43.0	29.7†
25,000–49,999	29.1	32.0	28.7	30.7	29.0
50,000–74,999	17.3	14.2	17.7	12.2	17.8
≥75,000	22.7	15.8	23.7	14.2	23.6
Smoking status (%)					
Current smokers	12.8	10.1	13.3†	9.8	13.1*
Former smokers	36.6	46.6	35.1	40.0	36.3
General health characteristics					
Diabetes (%)	12.4	19.5	11.3†	18.2	11.8†
Statin use (%)	16.4	26.3	14.7†	26	15.4†
Antihypertensive use (%)	32.6	48.9	30.1†	47.8	31.1†
Body-mass index (kg/m^2^)	28.3 ± 5.5	28.5 ± 4.9	28.3 ± 5.5	28.9 ± 5.6	28.3 ± 5.4†
Systolic blood pressure (mm Hg)	126 ± 21	135 ± 22	125 ± 21†	135 ± 23	125 ± 21†
HDL cholesterol (mg/dL)‡	51.0 ± 14.8	48.8 ± 13.6	51.3 ± 14.9†	52.0 ± 15.1	50.9 ± 14.7
Total cholesterol (mg/dL)‡	194.4 ± 35.9	195.2 ± 38.2	194.1 ± 35.4	193.5 ± 37.9	194.5 ± 35.6
Estimated glomerular filtration rate	78.1 ± 16.3	70.9 ± 16.5	79.2 ± 16.0†	69.9 ± 16.8	78.9 ± 16.0†
C-reactive protein (mg/L)	1.9 (3.4)	2.0 (3.2)	1.9 (3.4) *	2.2 (3.5)	1.8 (3.4) *
Physical activity (MET-min/wk)	4043 (5535)	3480 (4890)	4151 (5689) †	3814 (4668)	4073 (5703) †
Air pollution in year 2000					
Household PM_2.5_ (μg/m^3^)	16.8 ± 2.9	16.8 ± 2.9	16.8 ± 2.9	16.8 ± 2.9	16.8 ± 2.9
Household NO_x_ (ppb)	51.3 ± 27.8	51.8 ± 29.0	51.2 ± 27.8	51.0 ± 27.9	51.3 ± 27.9
Individual-level PM_2.5_ (μg/m^3^)	11.0 ± 3.3	11.0 ± 3.3	11.0 ± 3.3	10.9 ± 3.2	11.0 ± 3.3

Results are reported as percent, mean ± standard deviation or median (interquartile range)

*P*-values for continuous variables were calculated using *t* test with equal variances or Kolmogorov-Smirnov test where appropriate, and for categorical variables using chi-square test

† *P* < 0.001; *P < 0.05

‡ To convert total and HDL-C cholesterol from mg/dl to mmol/L, divide by 38.67

Outdoor concentrations of pollutants for the overall study population in year 2000 were on average (SD) 16.8 (2.9) μg/m^3^ for outdoor PM_2.5_, 11.0 (3.3) μg/m^3^ for individual-level PM_2.5_ and 51.3 (27.8) ppb for outdoor NO_x_ (Table 1).

**Fig. 2:**
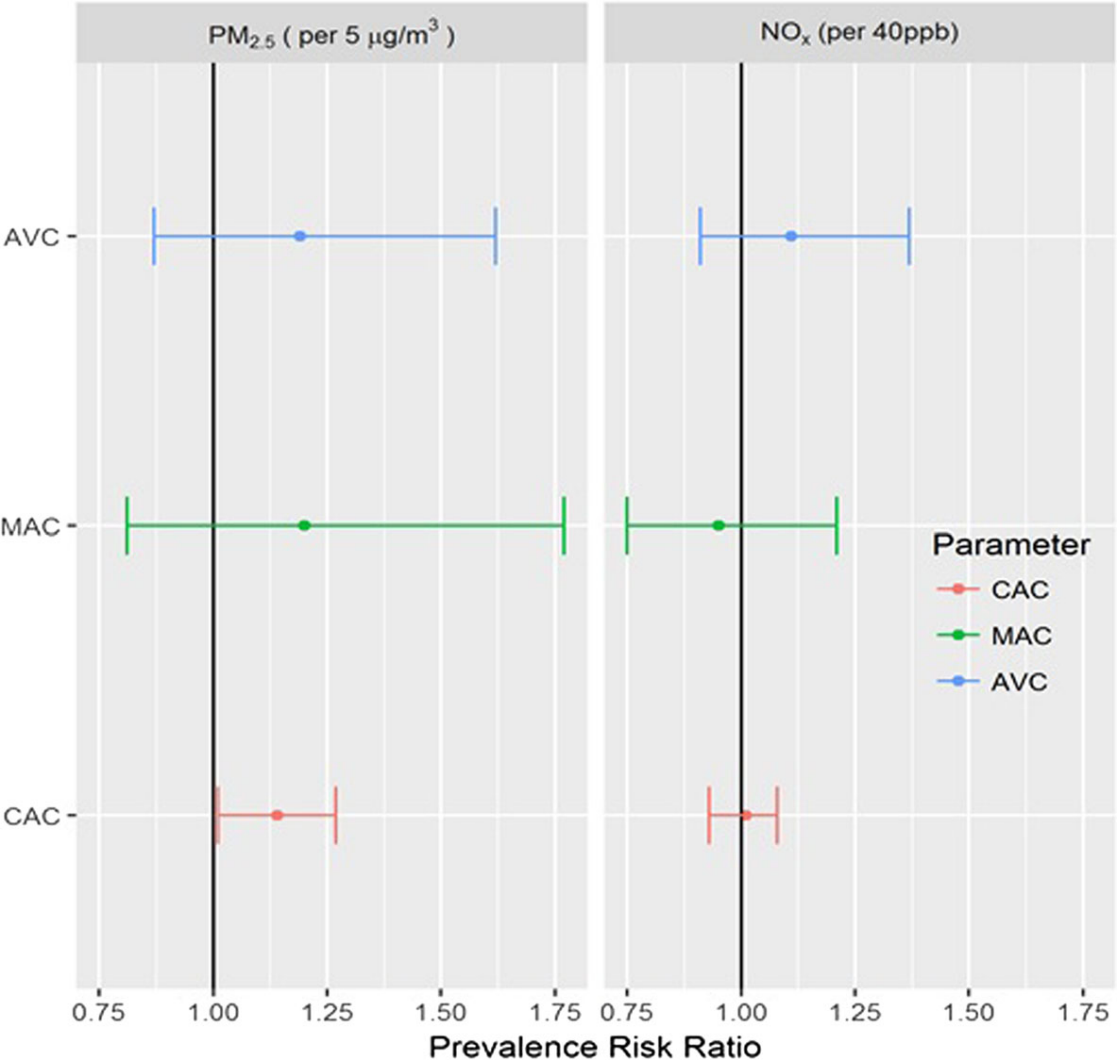
Adjusted* associations (with 95% CI) of air pollutants with prevalent AVC and MAC, compared to CAC, at the MESA baseline exam (2000–2002) *Adjusted for Model 3 covariates.

The prevalence of AVC and MAC at baseline was 13% for AVC and 9.4% for MAC, compared to 49.8% for CAC (Fig. 1). Among those with prevalent AVC, 28.7% had MAC > 0 at baseline. Conversely, among those with prevalent MAC, 40% had AVC > 0 at baseline. The adjPRR of AVC and MAC for each 5 μg/m^3^ higher outdoor PM_2.5_ concentration was 1.19 (95% CI 0.87, 1.62) and 1.20 (95% CI 0.81, 1.77), respectively (Fig. 2; Table 2, Model 3). The corresponding adjPRR for CAC was similar but reached statistical significance (Table 2). The adjPRR of AVC and MAC for each 40 ppb higher outdoor NO_x_ concentration was 1.11 (95% CI 0.91, 1.37) and 0.95 (95% CI 0.75, 1.21), respectively (Fig. 2; Table 2, Model 3). The corresponding adjPRR for CAC was similar and also not significant.

**Table 2:** Adjusted associations of air pollutants with prevalent AVC and MAC, compared to CAC at the MESA baseline exam (2000–2002)

Prevalence Risk Ratio (95% CI)			
Model	PM_2.5_ (per 5 μg/m^3^)	PM_2.5iwa_ (per 5 μg/m^3^)	NO_x_ (per 40 ppb)
Prevalent AVC			
1†	1.13 (0.85, 1.50)	1.23 (0.98, 1.54)	1.14 (0.94, 1.38)
2‡	1.18 (0.87, 1.60)	**1.27 (1.00, 1.62)**	1.13 (0.92–1.37)
**3‖**	1.19 (0.87, 1.62)	1.26 (0.98, 1.61)	1.11 (0.91–1.37)
4**	1.09 (0.82, 1.60)	**1.28 (1.01, 1.64)**	1.09 (0.86–1.33)
Prevalent MAC			
1†	1.26 (0.89, 1.78)	1.10 (0.83, 1.45)	1.02 (0.82–1.27)
2‡	1.20 (0.81, 1.76)	1.06 (0.79, 1.42)	0.94 (0.75–1.19)
**3‖**	1.20 (0.81, 1.77)	1.01 (0.75, 1.37)	0.95 (0.75–1.21)
4**	1.12 (0.75, 1.68)	1.02 (0.75, 1.38)	0.98 (0.72–1.20)
Prevalent CAC			
1†	**1.12 (1.01, 1.25)**	1.02 (0.94, 1.11)	1.03 (0.96, 1.10)
2‡	**1.13 (1.01, 1.27)**	1.04 (0.95, 1.13)	1.00 (0.93, 1.08)
**3‖**	**1.14 (1.01, 1.27)**	1.05 (0.96, 1.15)	1.01 (0.93, 1.08)
4**	**1.16 (1.03, 1.31)**	1.07 (0.98, 1.17)	1.01 (0.93, 1.09)

*Bolded results are statistically significant

†Model 1: age, sex, race/ethnicity, metropolitan area and CT scan type

‡Model 2: Model 1 plus smoking status, second-hand smoke exposure, physical activity, body mass index, total cholesterol, HDL cholesterol and statin use

‖Model 3 (Main model): Model 2 plus income and education

**Model 4: Model 3 plus family history of heart attack, systolic blood pressure, diabetes, anti-hypertensive medication use, C-reactive protein and eGFR

**Fig. 3:**
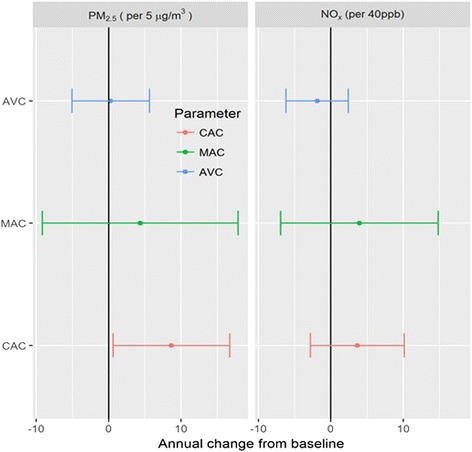
Adjusted* associations (with 95% CI) of annual averages of PM_2.5_ and NO_x_ with annual change in AVC and MAC over follow-up, compared to CAC *Adjusted for Model 3 covariates.

Over a mean follow-up of 2.5 years, the mean (standard deviation) annual change (in Agatston units/year) for AVC was 2.0 (38.9) and for MAC was 8.4 (97.2) (Fig. 1). For every 5 μg/m^3^ higher outdoor PM_2.5_ concentration, the mean change in Agatston units/year from baseline was 0.29 (95% CI: -5.05, 5.63) for AVC and 4.38 (95% CI: -9.13, 17.88) for MAC (Fig. 3; Table 3, Model 3). For each 40 ppb higher in outdoor NOx, the average change from baseline was −1.90 (95% CI: -6.20, 2.40) and 3.94 (−6.93, 14.83) Agatston units/year for AVC and MAC respectively (Fig. 3; Table 3, Model 3). The corresponding annual change in CAC are also presented in Fig. 3 and Table 3 **.** The associations for CAC were somewhat stronger and statistically significant, although confidence intervals overlapped with the findings for MAC.

**Table 3:** Adjusted associations of annual averages of PM_2.5_ and NO_x_ with annual change in AVC and MAC over follow-up, compared to CAC

Results presented as Agatston units/year (95% CI)			
Model	PM_2.5_ (per 5 μg/m^3^)	PM_2.5iwa_ (per 5 μg/m^3^)	NO_x_ (per 40 ppb)
Change in AVC			
1†	0.99 (−3.77, 5.75)	−0.37 (−3.89, 3.16)	−0.73 (−4.53, 3.06)
2‡	0.33 (−4.98, 5.64)	−0.21 (−4.08, 3.66)	−1.74 (−5.94, 2.46)
**3‖**	0.29 (−5.05, 5.63)	−0.42 (−4.37, 3.54)	−1.90 (−6.20, 2.40)
4**	−0.78 (−5.50, 3.94)	−1.15 (−4.55, 2.25)	−2.58 (−6.30, 1.20)
Change in MAC			
1†	2.85 (−9.21, 14.91)	−0.61 (−8.94, 7.71)	1.28 (−8.33, 10.89)
2‡	3.40 (−10.03, 16.83)	1.28 (−7.82, 10.38)	2.32 (−8.29, 12.93)
**3‖**	4.38 (−9.13, 17.88)	2.15 (−7.14, 11.43)	3.94 (−6.93, 14.83)
4**	2.72 (−11.21, 16.65)	1.44 (−8.24, 11.12)	2.26 (−8.87, 13.39)
Change in CAC			
1†	6.84 (−0.68, 14.36)	2.64 (−2.70, 7.98)	3.44 (−2.56, 9.43)
2‡	**8.36 (0.36, 16.37)**	3.18 (−2.43, 8.79)	3.43 (−2.89, 9.75)
**3‖**	**8.66 (0.61, 16.71)**	3.07 (−2.65, 8.79)	3.66 (−2.82, 10.14)
4**	**9.12 (1.16, 17.09)**	3.39 (−2.16, 8.94)	2.55 (−3.31, 8.91)

*Bolded results are statistically significant

†Model 1: age, sex, race/ethnicity, metropolitan area and CT scan type

‡Model 2: Model 1 plus smoking status, second-hand smoke exposure, physical activity, body mass index, total cholesterol, HDL cholesterol and statin use

‖Model 3 (Main model): Model 2 plus income and education

**Model 4: Model 3 plus family history of heart attack, systolic blood pressure, diabetes, anti-hypertensive medication use, C-reactive protein and eGFR

In secondary analyses, we also present the results of the individually-weighted PM2.5 exposure (PM_2.5iwa_) for cross-sectional (Table 2) and longitudinal (Table 3) analyses. Associations were somewhat stronger for AVC prevalence (Table 2) but weaker for AVC/MAC/CAC progression analysis (Table 3). In sensitivity analysis, we also examined the effect of each pollutant as adjusted for the other co-pollutant on AVC and MAC, and the results remained largely unchanged from the primary analysis of single exposure modules. Also, there was no evidence for effect modification when pollutants were combined in the same model. Additionally, there were no significant interactions by age, sex or race/ethnicity for the associations tested.

## Discussion

In this ethnically diverse population free of preexisting clinical CVD, there was a trend towards increased 2.5-year progression of MAC with exposure to outdoor PM_2.5_. However, this association could not be confirmed, although we did confirm an adverse association of PM_2.5_ with the more prevalent CAC even over this short duration.

To our knowledge, this is the first study to investigate the association between ambient air pollution and valvular calcification. Kaufman et al. reported a significant association of long-term (a 10-year) exposure to PM_2.5_ with CAC progression in this same cohort [9], and we also confirmed that this association with CAC remains significant even after considering a short follow-up period of 2.5 years.

In contrast to AVC, the estimate for the association of PM_2.5_ with the 2.5-year progression of MAC was similar in magnitude to CAC (Fig. 2). These findings are consistent with prior reports of a much stronger association of CAC with MAC than with AVC [13]. It is therefore possible that the same biological mechanisms may underlie both diseases [8]. The findings herein would suggest that the association of air pollution is specific with CAC rather than with vascular calcification in general. However our study population had a low prevalence of MAC, which may have reduced statistical power. For comparison, in the Cardiovascular Health Study, an older population (mean age 72 ± 5 years), the prevalences of AVC and MAC were 59% and 41% respectively [28]. Future studies evaluating the associations of PM_2.5_ with AVC and MAC in cohorts with higher prevalence of valvular calcification would likely provide further insight.

Furthermore, given that the exposures in this cohort were low by historical and international standards exposures [31], it is also possible that there is a lower threshold for atherosclerosis progression in the coronary arteries resulting from exposure to ambient PM_2.5_ compared to calcification of left-sided heart valves. Additionally, the left-sided heart is a relatively high flow area compared to coronary arteries and may take longer for progressive calcium deposition to build up. This implies a longer follow-up period may be needed to better assess the associations of air pollution with AVC and MAC. Although CAC and valvular calcification share many risk factors in common and are correlated with each other [13], there still may remain differences in the risks conferred by traditional and non-traditional CVD risk factors across the various vascular beds.

Although our findings regarding air pollution and AVC and MAC are consistent with no association, our data are novel in that we evaluated the impact of air pollution with progression of calcification in vascular beds previously not extensively studied in a large population-based cohort free of CVD. Our findings have relevance to public health globally, as Fox et al. previously reported the independent association of MAC with incident CVD outcomes and death [17]. Our findings of a suggestive trend towards increased 2.5-year progression of MAC with exposure to outdoor PM_2.5_, if confirmed in additional well-powered studies with longer periods of follow-up, may provide insight into other potential underlying mechanisms linking air pollution to CVD morbidity and mortality.

This study was conducted in six U.S. cities with a racially/ethnically diverse population without prior clinical CVD at the time of enrollment, reducing the likelihood of confounding by poorer health status. AVC, MAC and CAC were assessed with serial CT scan measurements and potential confounders were also well characterized in MESA. Furthermore, concentrations of pollutants were estimated using state-of-the-art methods that enabled prediction of household and individual level exposures. Nonetheless, there are some limitations to this study that must be acknowledged. First, concentrations of pollutants during follow-up of this cohort may not be high enough to capture the association between air pollution and progression of valvular calcification. The mean household-level PM_2.5_ concentration for this cohort over the follow-up period was 15.3 (range: 8.8–26.5) μg/m^3^. Compared to the European Union’s annual PM_2.5_ standards of 25 μg/m^3^, the levels measured in this study are much lower [31]. Hence, findings for AVC and MAC cannot be generalized to other industrializing countries with considerably higher air pollution levels [32, 33]. Second, although a significant association between PM_2.5_ and CAC was found even after 2.5 years of follow-up, this timeframe was likely too short to assess associations of air pollution with AVC and MAC, which would relatively be slower possibly because of differences in hemodynamics between the left heart chambers and the coronary arteries.

## Conclusions

In summary, an adverse trend was found between outdoor PM_2.5_ and 2.5-year progression of MAC in a racially/ethnically diverse cohort from 6 U.S. cities with low exposures by historical and international standards, although this association could not be confirmed. On the other hand, we did confirm the association of PM_2.5_ with the more prevalent CAC over this same short time period. Additional well-powered studies with long periods of follow-up and in communities with higher levels of exposures are warranted to better assess the association between air pollution and valvular calcification.

## Abbreviations

adjPRR: Adjusted prevalence risk ratio
AVC: Aortic valve calcium
BMI: Body Mass Index
CAC: Coronary artery calcium
CI: Confidence Intervals
CT: Computed Tomography
CRP: C-Reactive Protein
CVD: Cardiovascular Disease
EPA: Environmental Protection Agency
eGFR: Estimated Glomerular Filtration Rate
HDL: High Density Lipoprotein
MAC: Mitral annular calcium
MESA: Multi-Ethnic Study of Atherosclerosis
NO_x_: Oxides of nitrogen
PM_2.5_: Ambient particulate matter <2.5 μm
